# Acoustic Neuroma Mimicking Orofacial Pain: A Unique Case Report

**DOI:** 10.1155/2016/1947616

**Published:** 2016-12-07

**Authors:** Praveenkumar Ramdurg, Naveen Srinivas, Vijaylaxmi Mendigeri, Surekha R. Puranik

**Affiliations:** ^1^Department of Oral Medicine and Radiology, PMNM Dental College and Hospital, Bagalkot, Karnataka, India; ^2^Department of Orthodontics, PMNM Dental College and Hospital, Bagalkot, Karnataka, India; ^3^Department of Oral Medicine and Radiology, PMNM Dental College and Hospital, Bagalkot, Bagalkot District, India

## Abstract

Acoustic neuroma (AN), also called vestibular schwannoma, is a tumor composed of Schwann cells that most frequently involve the vestibular division of the VII cranial nerve. The most common symptoms include orofacial pain, facial paralysis, trigeminal neuralgia, tinnitus, hearing loss, and imbalance that result from compression of cranial nerves V–IX. Symptoms of acoustic neuromas can mimic and present as temporomandibular disorder. Therefore, a thorough medical and dental history, radiographic evaluation, and properly conducted diagnostic testing are essential in differentiating odontogenic pain from pain that is nonodontogenic in nature. This article reports a rare case of a young pregnant female patient diagnosed with an acoustic neuroma located in the cerebellopontine angle that was originally treated for musculoskeletal temporomandibular joint disorder.

## 1. Introduction

Orofacial pain is the most common problem encountered by the patient that leads to frequent visits to the dental office, as it mimics vast array of disorders arising from orofacial structures, diseases due to generalized musculoskeletal, peripheral, or central nervous system, and psychological abnormality [1]. In some rare instances, the pain may also arise from neurogenic sources involving cranial nerves V [2, 3], VII [4], VIII [5], and IX [2].

Several reports showed that otologic [6] and ophthalmological [7] clinical manifestations mimic orofacial pain. Thus, well-trained dentists should be capable of diagnosing the source of these complaints before starting any dental treatment in order to avoid unnecessary interventions. One rare cause of orofacial pain is intracranial tumors [8]. According to Bullitt et al., about 1% of patients with orofacial pain will have intracranial tumors as the underlying cause [9]. These patients may undergo unnecessary dental interventions before the correct diagnosis is made.

Tumors of the VIII cranial nerve or acoustic neuroma (AN) is a relatively uncommon, benign, usually slow-growing tumor that develops from the vestibulocochlear nerve supplying the inner ear [10–12].

The Schwann cell sheath from which these tumors develop lies distal to the Glial-Schwann cell junction, which is usually located close to the point where the eighth nerve enters the internal auditory meatus. Consequently, AN arise almost invariably within the meatus itself but expand in a medial direction through the orifice of the meatus and into the potential space formed by the cerebellopontine angle. Here, their close proximity to the roots or proximal portions of various cranial nerves ultimately leads to the development of signs and symptoms due to the pressure on these nerves [13].

Compression of these structures may result in a series of complications with the most common symptoms being tinnitus, hearing loss, and postural imbalance [14]. Despite this, these tumors can also present with other symptoms like temporomandibular disorders (TMD), orofacial pain, numbness or tingling in the face, headache, dizziness, facial paresis, and trigeminal nerve disturbances [15].

To date, there are very few case reports in the dental literature of AN mimicking orofacial pain of nonodontogenic origin; hence, the purpose of this article was to report one such rare case of AN impersonating nonodontogenic orofacial pain.

## 2. Case Report

A 30-year-old female patient in her third trimester visited a dentist with a chief complaint of pain in the right side of maxilla and preauricular region in the month of April 2014. Pain was gradual in onset, moderate in intensity, and continuous in nature. The pain radiated to the temporal and auricular region of the same side. Examination of the patient revealed tenderness of the TMJ and muscles of mastication on the right side and bilateral attrition of maxillary and mandibular first molars. The intensity of pain was measured using visual analogue scale which was at 8. Correlating the history and clinical findings, a diagnosis of temporomandibular joint disorder secondary to bruxism was considered. Patient was recalled after 15 days with instructions to use night guard, restricted mouth opening, soft diet, and physiotherapy.

Patient reported reduced pain with VAS score of 4 after 20 days; hence, she was was advised to continue with the same instructions and constant follow-up.

Patient reported back to the clinic with aggravated symptoms in December 2015. The pain presented as shock-like that aggravated on brushing and washing face with added symptoms of tinnitus, hearing impairment in right ear, and intermittent loss of balance. Radiographic examination revealed no abnormality with TMJ and surrounding structures. Hence, patient was referred to ENT specialist and audiometry was performed and 48 dB of hearing loss was noted in audiometry test. A more serious pathology was suspected and computed tomography (CT) and magnetic resonance imaging (MRI) were requested. Since the patient was in pain at the time of the examination and the pain had a neuropathic quality and presentation, carbamazepine was initially prescribed for her, which decreased her intensity of pain.

**Figure 1:**
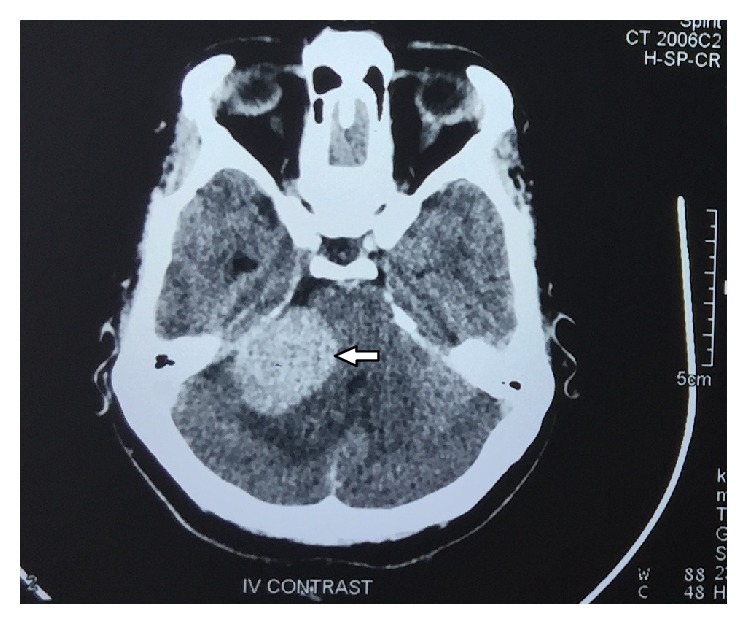
Contrast axial CT shows an ovoid homogeneous enhancing lesion (38 mm × 27 mm) involving right cerebellopontine cistern (white arrow).

**Figure 2:**
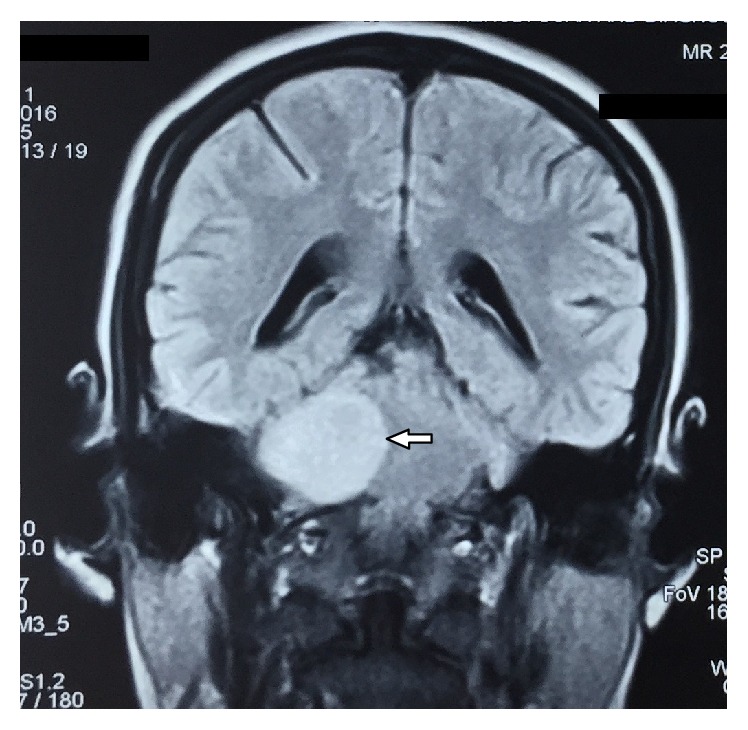
Contrast coronal MRI shows well-defined heterogeneously enhancing T1 hypo and T2 predominantly hyperintense lesion in the right cerebellopontine angle (white arrow).

**Figure 3:**
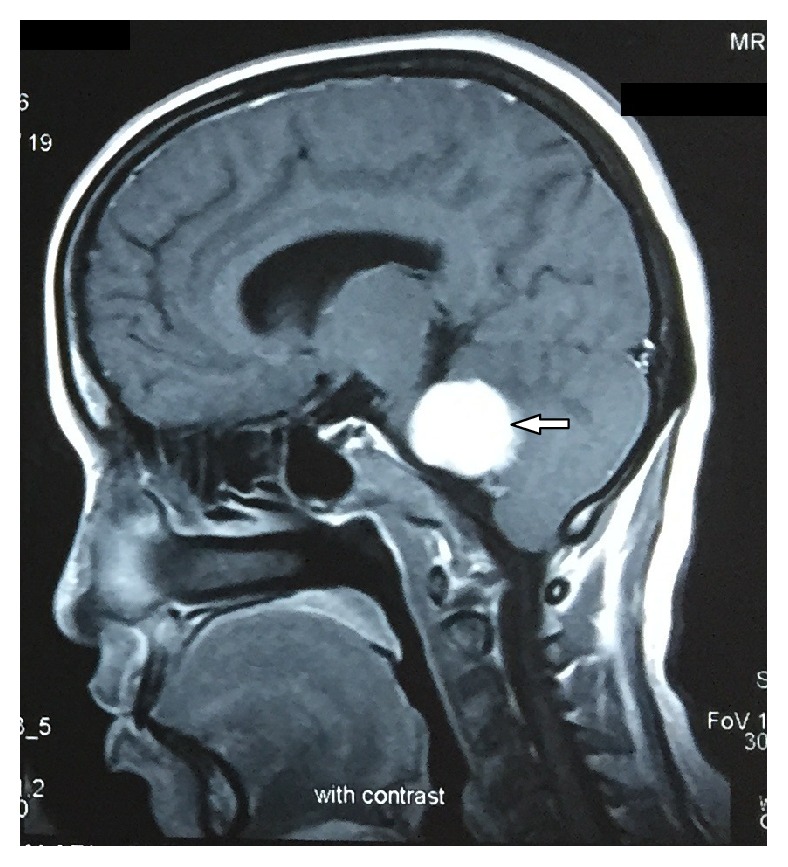
Contrast sagittal MRI shows well-defined heterogeneously enhancing T1 hypo and T2 hyperintense mass in the right cerebellopontine angle causing mild mass effect on the brain stem (white arrow).

**Figure 4:**
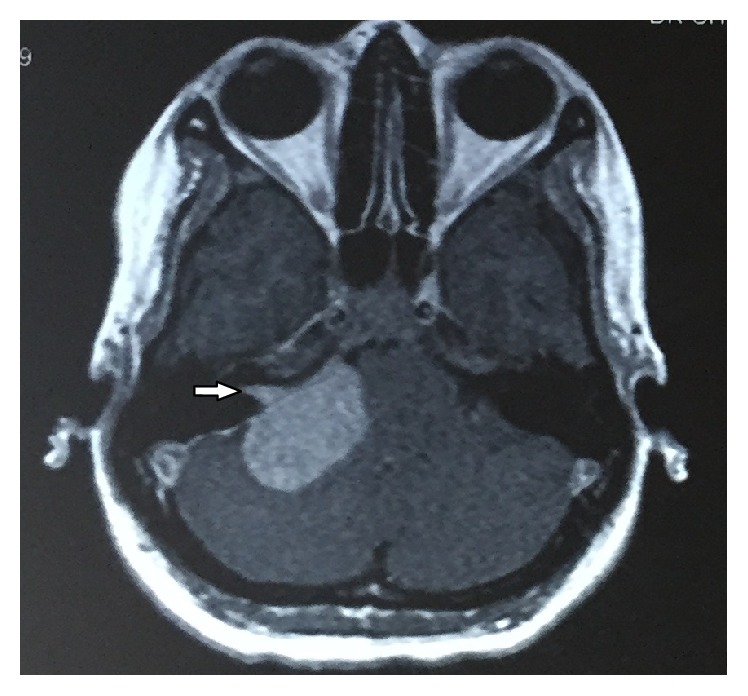
Contrast axial MRI shows the extension of the tumor into the internal auditory canal represents “shark fin” appearance (white arrow).

**Figure 5:**
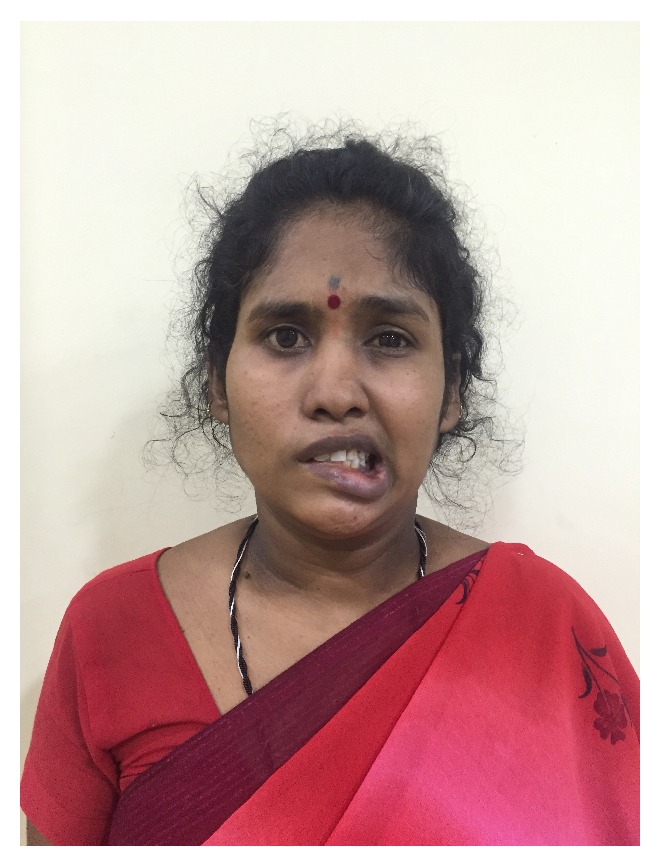
Postoperative image shows paralysis of face on right side.

**Figure 6:**
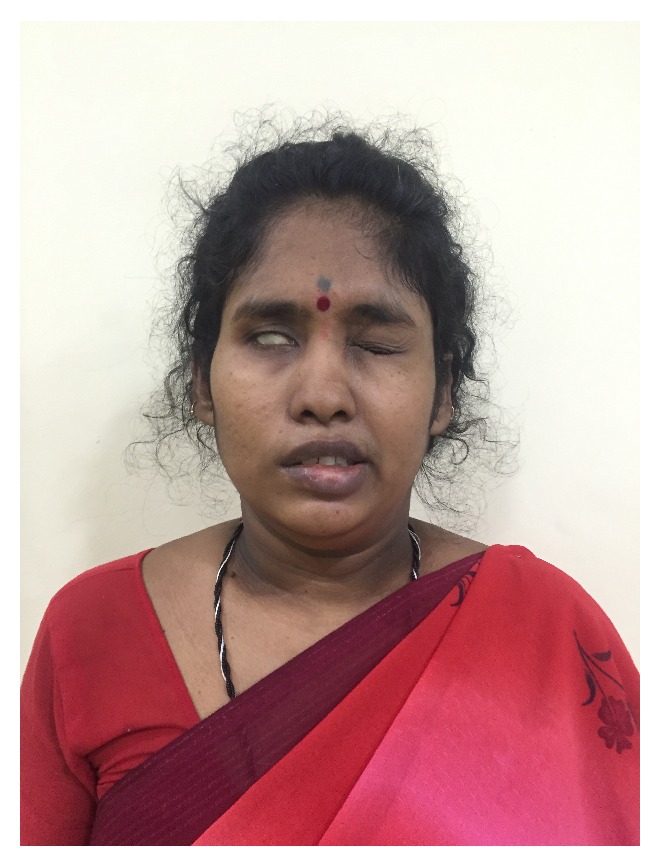
Postoperative image shows inability to close the right eyelid and upward rolling of right eyeball.

Contrast CT (Figure 1) report showed that an approximately 38 × 27 mm ovoid homogeneous enhancing extra axial mass lesion involving right cerebellopontine cistern was observed. Further contrast MRI revealed 25 × 37 × 31 mm well-defined heterogeneously enhancing T1 hypo and T2 predominantly hyperintense space occupying mass lesion in the right cerebellopontine angle cistern causing mild mass effect on the brain stem with extension to right internal auditory canal with subtle canal widening suggestive of acoustic neuroma (Figures 2 and 3). The extension of the tumor into the internal auditory canal represented “shark fin” appearance (Figure 4). Immediately, the patient was referred to a neurosurgeon to evaluate the lesion. Right retromastoid suboccipital craniotomy and total excision of lesion were performed leading to damage of cranial nerves VII and VIII resulting in hearing loss and facial paralysis with loss of taste sensation on the affected side (Figures 5 and 6).

**Figure 7:**
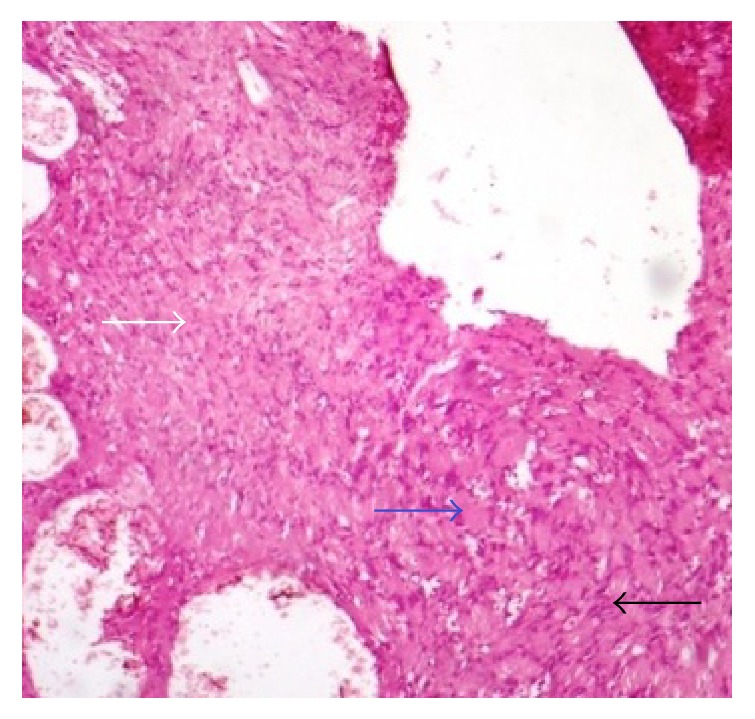
Histological features (H&D ×40) show Antoni-A areas (black arrow), Antoni-B areas (white arrow), and Verocay bodies (blue arrow).

**Figure 8:**
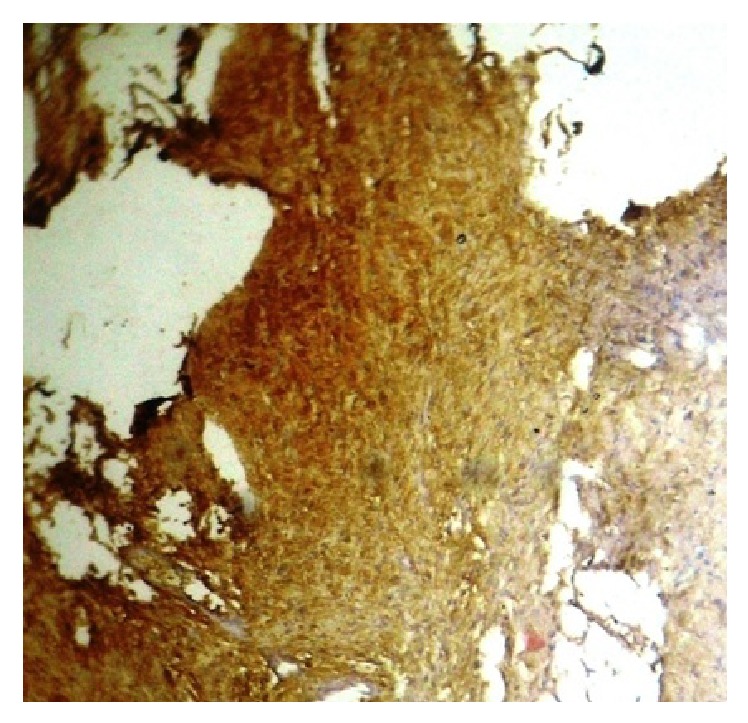
Immunohistochemistry (×40) positive for S-100.

Histological analysis of the lesion revealed two microscopic patterns in varying amounts. Few areas showed streaming fascicles of spindle shaped Schwann cells (Antoni-A areas). These spindle shaped cells were arranged in a palisaded manner around acellular, eosinophilic areas (Verocay bodies). In other areas, the spindle shaped cells revealed relatively less cellular and less organized areas within a myxomatous stroma (Antoni-B areas). Few blood vessels showed hemorrhage and fibrin within the lumen. Immunohistochemical analysis revealed that the lesion was positive for S-100 protein (Figures 7 and 8).

## 3. Discussion

A complete history, clinical examination, and through diagnostic work-up ruled out an odontogenic cause for the patient's pain. The absence of work, family, and personal conflicts excluded psychological pain. Initially, the clinical signs of bruxism that is attrition of posterior molars in maxilla and mandible along with the tenderness of the right TMJ tilted the diagnosis towards musculoskeletal-TMD. Bite guard, physiotherapy, and other instructions reduced the pain but the patient did not return for follow-up and further treatment because of pregnancy. After one and half years, the patient reported advanced clinical features and AN, and diagnosis was made based on MRI imaging.

The AN originates from the Schwann cell, in the peripheral portion of superior and inferior vestibular nerves, and also from cochlear nerve [16]. The AN occurs in an incidence of about 1 : 100000 inhabitants per year [17]. In most recent statistics, an increase of such incidence has been reported due to frequent use of more sensitive magnetic resonance (MR) techniques, diagnosing very small tumors [18]. It happens independently of ethnicity and is more frequently diagnosed in men within 50–60 years of age (61%) [5]. Orofacial pain as the sole symptom of an intracranial tumor is rare. When orofacial pain is caused by such a lesion, neurological abnormalities are usually present [19].

In the present case, initial clinical features suggested orofacial pain but late stages exhibit neurological abnormalities like trigeminal neuralgia. Although the patient exhibited the classic symptoms of acoustic neuroma, namely, tinnitus and loss of hearing, the patient's primary concern was neuralgic pain that became unbearable. These symptoms had only been diagnosed as part of the patient's dental follow-up. Upon limited success of treatment of orofacial pain and considering her young age, the patient was referred for MRI suspecting an intracranial tumor, which led to the diagnosis of trigeminal neuralgia secondary to acoustic neuroma.

Few case reports [5, 9, 20] were published with a confirmed diagnosis of AN mimicking orofacial pain, whereas most of the cases [21–23] in the literature are of trigeminal neuralgia secondary to acoustic neuroma. In our case, the patient manifested features of orofacial pain in early visits but in late stage clinical features suggested trigeminal neuralgia. This may be because some authors believe that as tumor size increases it pushes the trigeminal nerve root against the superior cerebellar artery, producing a neurovascular conflict similar to the vascular compression theory proposed for classic trigeminal neuralgia. Another school of thought suggests that the increasing pressure on the trigeminal root or ganglion may induce loss of myelination in the trigeminal sensory root resulting in ephaptic short-circuiting within the nerve root, which results in facial pain and sensory deficits [2].

MRI images of AN in the literature [24] show “trumpeted internal acoustic meatus sign” or “ice cream cone sign,” which is a distinguishing feature between AN and other cerebellopontine angle entities. In our case, extension of the AN has a typical “shark fin” like appearance.

Early diagnosis of a vestibular schwannoma is key to preventing its serious consequences. There are three options for managing a vestibular schwannoma: observation, radiation, and surgical removal [25]. In the present case, right retromastoid suboccipital craniotomy and total excision of lesion were done. Surgical access to this confined zone is difficult; there is a high likelihood of introducing new symptoms or exacerbating preexisting conditions. In our case, the patient developed facial paralysis, loss of taste sensation, and hearing loss on the right side. Facial paralysis and loss of taste sensation are due to damage to the facial nerve which resulted in reduced tonicity in the muscles of facial expression, as well as affecting taste in the anterior two-thirds of the tongue via the chorda tympani nerve. Due to encirclement of the vestibule-cochlear nerve around the tumor,, the patient suffered hearing loss on the right side. Literature [26] shows that about 68% of AN patients are diagnosed by otolaryngologists, 28% are diagnosed by neurosurgeons, 19% are diagnosed by neurologists, 5% are diagnosed by physicians, and less than 5% are diagnosed by dentists, a very low percentage.

## 4. Conclusion

Hence, the dentist should give more emphasis through history and clinical and radiological examination and also include AN in the differential diagnosis when considering orofacial pain, TMD, and trigeminal neuralgia in young age.
